# Efficient Detection of Longitudinal Bacteria Fission Using Transfer Learning in Deep Neural Networks

**DOI:** 10.3389/fmicb.2021.645972

**Published:** 2021-06-08

**Authors:** Carlos Garcia-Perez, Keiichi Ito, Javier Geijo, Roman Feldbauer, Nico Schreiber, Wolfgang zu Castell

**Affiliations:** ^1^Information and Communication Technology Department (ICT), Complex Systems, Helmholtz Zentrum München, Neuherberg, Germany; ^2^Department of Microbiology and Ecosystem Science, University of Vienna, Vienna, Austria; ^3^Functional and Evolutionary Ecology, University of Vienna, Vienna, Austria; ^4^Department of Mathematics, Technische Universität München, Munich, Germany

**Keywords:** bacteria division, longitudinal bacterial fission, bacteria classification, deep learning, transfer learning, image processing, image segmentation

## Abstract

A very common way to classify bacteria is through microscopic images. Microscopic cell counting is a widely used technique to measure microbial growth. To date, fully automated methodologies are available for accurate and fast measurements; yet for bacteria dividing longitudinally, as in the case of *Candidatus* Thiosymbion oneisti, its cell count mainly remains manual. The identification of this type of cell division is important because it helps to detect undergoing cellular division from those which are not dividing once the sample is fixed. Our solution automates the classification of longitudinal division by using a machine learning method called residual network. Using transfer learning, we train a binary classification model in fewer epochs compared to the model trained without it. This potentially eliminates most of the manual labor of classifying the type of bacteria cell division. The approach is useful in automatically labeling a certain bacteria division after detecting and segmenting (extracting) individual bacteria images from microscopic images of colonies.

## 1. Introduction

Bacterial cell shapes can vary from cocci and rods to more exotic shapes such as spirals or branches (Kysela et al., 2016). Diverse activities influence the bacterial shape such as division, or adaptations to local physical constraints. Microscopy approaches are commonly used to observe and classify different microorganisms according to their different morphological features. Microscopic cell counting is one of the most common techniques used to measure microbial growth. This approach usually relies on automatic microscopic cell counting using digital image analysis software in order to determine division rates (Daims et al., 2006; Nekrasov et al., 2013). Longitudinal bacterial division (or fission) is a rare feature among bacteria (Pende et al., 2018). Thus, discriminating between perpendicular and longitudinal division requires novel approaches in image analysis to differentiate those cells undergoing a division, whereby they widen instead of elongating.

### 1.1. Convolutional Network

This is what is called a binary classification problem. Our contribution is in using machine learning (ML) to automatically classify the two types of bacterial division (i.e., longitudinal and non-longitudinal) from microscopic images of the bacteria. It has a potential to substantially alleviate manual classification and counting of exotic cell splits, just like the automatic hand digit recognition system did to the postal services. ML is a term that refers to algorithms that model a relationship or a map *f* between input *x* and output *y* such as in *y* = *f*(*x, w*) from given sets of data. If this model is determined from multiple examples, say *N* input–output pairs [*x*_*i*_, *y*_*i*_], *i* ∈ 1, 2, ⋯ , *N*, it is called supervised learning. Specifically, the array of model parameters *w*, often called weights in artificial neural network literature, is optimized (i.e., learned) by minimizing some error measures calculated from the discrepancy between target output value *y*_*i*_ and predicted ŷ = *f*(*x*_*i*_, ŵ), where ŵ is an estimate of the optimal model parameters *w*. This process of searching for the optimal *w* through the data is called training and requires an optimization algorithm. The training normally includes a validation and test process in which data not used in generating ŵ are used to compute the error. This is done to estimate the prediction accuracy with the hitherto unseen data and serves as stopping criteria for the training.

In our case, the data consist of individual bacteria images (i.e., instances of *x*). For each of the images, we have a corresponding label (i.e., instances of *y*) to identify whether it is a longitudinal division or not. With the data, we want to train a model *f*. Using Python as the programming language, we can use existing codes in PyTorch (Paszke et al., 2019) to perform the ML task of training *f*. That is, a selection of data reading function, the model *f*, and optimization algorithms to obtain ŵ are furnished by PyTorch.

**Figure 1:**
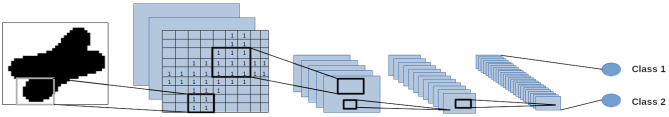
Convolutional neural network (CNN) 2D architecture. A stack of convolutional layers precedes the fully connected neural network architecture. Each set of blue squares represent a layer of the network. In the first layer, the network learns some features of the input image, which then passes a feature map to the next layer to learn new features, and so on.

In particular, we use Residual Network (ResNet) (He et al., 2016), one of the state-of-the-art deep learning (DL) architectures in image classification. An important advancement in the network architecture was due to the convolutional neural network (CNN) (LeCun et al., 1998). CNN learns an appropriate set of kernels (each having a set of weights for the pixels in a square region) that swipe the image from left to right, top to bottom shifting at a constant stride of pixels (Figure 1). The kernel performs an inner product with the receptive region in the input image to generate an output pixel and the resulting 2D image is called the feature map. In essence, a kernel creates 2D image features. Convolutional layers can be stacked one after the other to learn increasingly complex features as the layer goes deeper from the input layer. Placing the convolutional layer before the traditional fully connected layer enables the automatic learning of features effective for the task of image classification. CNN also has pooling layers that downsample the input image to smaller dimensions.

To illustrate the convolution layer (Figure 1), let us suppose we have a 4 by 4 pixel image like the following matrix.


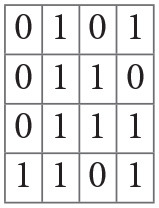


Then, suppose we sweep a 2 by 2 kernel, with weights denoted as *a*, *b*, *c*, and *d*,


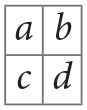


on the image at a stride of one pixel. At each position, an inner product is calculated. For example, in the first position at upper left corner of the image, we have

$$\langle \left(\begin{array}{cc} 0 & 1\\ 0 & 1 \end{array}\right),\left(\begin{array}{cc} a & b\\ c & d \end{array}\right)\rangle =0\cdot a+1\cdot b+0\cdot c+1\cdot d=b+d.$$

Next, we move the kernel one pixel to the right and compute

$$\langle \left(\begin{array}{cc} 1 & 0\\ 1 & 1 \end{array}\right),\left(\begin{array}{cc} a & b\\ c & d \end{array}\right)\rangle =1\cdot a+0\cdot b+1\cdot c+1\cdot d=a+c+d.$$

Likewise, the last position of the kernel in the first row generates, 0 · *a* + 1 · *b* + 1 · *c* + 0 · *d* = *b* + *c*. The next kernel position goes one pixel downward and starts again from the leftmost position, 0 · *a* + 1 · *b* + 0 · *c* + 1 · *d* = *b* + *d*.

We continue the process until the kernel reaches the bottom right corner. In this example, we obtain a feature map of 3 by 3 as in the following matrix.


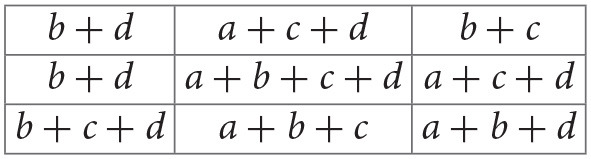


The actual values of the kernel weights *a, b, c, d* are learned during the training. Depending on the values of weights, different features can be extracted from the same image. Thus, each feature map is a consequence of a particular instance of kernel weights.

### 1.2. Residual Network

**Figure 2:**
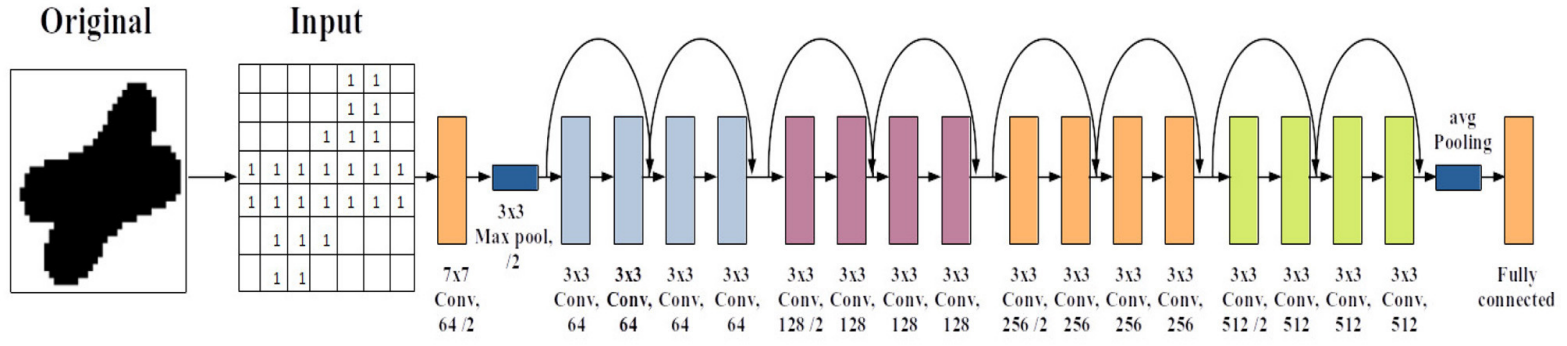
ResNet-18 architecture. In addition to the convolutional layer stacking, it has forward skipping connections that is added to the output of every few layers that enables a “residual” learning.

CNN substantially reduced the number of weights (i.e., model parameters) to be trained while also extracting topological information or features of 2D images more efficiently compared to the conventional feed-forward deep neural network (DNN) with vectorized inputs. Deep networks show increasing power in learning complex patterns with the number of hidden layers inside the network. However, optimizing weights in very deep networks becomes more difficult at the same time. The advent of ResNet mitigated this limitation. ResNet employs a so-called “identity shortcut connection” that skips one or more layers (see Figure 2). This creates network paths of different depths, which in essence form an ensemble of shallower models that are trained simultaneously (Veit et al., 2016). This made ResNet very accurate and easier to train compared to the classical CNN.

To see the reasoning behind the skipping connection in ResNet, consider learning a function *h*:*X* → *Y*, or

(1) $$\begin{array}{r} y=h\left(x,w\right) \end{array}$$

from many instances of input *x* and output *y* by tuning the weights *w*. Let us assume *X* and *Y* have the same dimension and represent the input and output space of certain hidden layer(s) of a neural network. The problem in DL is that you run into a situation where input and output becomes similar or very small in magnitude (*y* ≃ *x*) as the number of layer becomes larger. Beyond certain number of layers, the numerical inaccuracies outweigh the benefit of added complexity of the model even when trained with abundant data. He et al. (2016) reasoned that it would be easier to learn the residual *f*(*x, w*) = *h*(*x, w*) − *x* rather than Equation (1). That is, we subtract input *x* from both sides of Equation (1) to obtain

(2) $$\begin{array}{r} y-x=h\left(x,w\right)-x \end{array}$$

(3) $$\begin{array}{r} =f\left(x,w\right). \end{array}$$

This shows that *f*(*x, w*) represents only the difference (or the “residual”) between input *x* and output *y*. This gives

(4) $$\begin{array}{r} y=x+f\left(x,w\right). \end{array}$$

Thus, the skipping bridge in the ResNet diagram in Figure 2 realizes (Equation 4) every few layers (e.g., every two layers in the figure). He et al. (2016) also reasoned that if a layer was insignificant, the formulation as in Equation (4) would more easily drive *w* to “zero” and establish the relation

y=x.

Formulating the stacking of feature learning layers as learning of “residuals” is rather Copernican. However, the idea of capturing the residual separately and using it for compensating for the loss of accuracy is not new. It has been in use as a technique to avoid the cancellation of significant digits. For example, consider a rather common case where one would like to compute a large sum of floating-point numbers *x*_*k*_, where *k* ∈ {1, 2, 3, ⋯ , *N*} is an iteration counter and *N* is possibly very large. One would normally do a for-loop of the following recursive equation:

(5) $$\begin{array}{r} S_{k+1}=S_{k}+x_{k}. \end{array}$$

However, one soon faces a situation in which the partial sum *S*_*k*_ does not get updated or accumulates a substantial error in the resulting total sum *S*_*N*_. Then, a possible remedy is to set a residual term *R*_*k*_ in the recursive equation of the partial sum. Thus, the for-loop runs the following. Setting *T* and *U* as dummy variables and initializing *S*_1_ = *R*_1_ = 0,

(6) $$\begin{array}{r} T=S_{k} \end{array}$$

(7) $$\begin{array}{r} U=x_{k}+R_{k} \end{array}$$

(8) $$\begin{array}{r} S_{k+1}=S_{k}+U \end{array}$$

(9) $$\begin{array}{r} R_{k+1}=U-\left(S_{k+1}-T\right) \end{array}$$

Equation (7) is analogous to Equation (4). The residual term *R*_*k*_ keeps a record of the errors and corrects for the cumulative discrepancy. The above method is known to produce double-precision-like results even if computed in single precision (Iri and Fujino, 1985, p.18).

### 1.3. Related Work

Among the many areas where computer vision is applied, health is a very important one (Yadav and Jadhav, 2019; Sharma et al., 2020). Training a CNN from scratch requires a large amount of labeled data as well as high computational power. To overcome this challenge, the knowledge of a previously trained CNN model can be transferred to train new data with similar features. This technique is known as transfer learning.

Transfer learning (Pan and Yang, 2010) consists of passing previous knowledge to classify new objects. For example, we can transfer the knowledge of the shape of an object to another similar one. This reduces the learning time for the new object. The use of transfer knowledge has been successfully applied in the classification of X-ray images (Yadav and Jadhav, 2019; Rahman et al., 2020; Sharma et al., 2020). Moreover, in bacteria classification we also find the application of transfer learning as in Buetti-Dinh et al. (2019), Lin et al. (2019), Treebupachatsakul and Poomrittigul (2019), and Talo (2019). Nevertheless, for the classification of longitudinally dividing bacteria, we have not found any work or publication to our knowledge. Encouraged by this gap, we decided to apply a deep learning approach to solve this type of problem. Despite these advancements, the quality of the trained model is strongly influenced by the number and quality of data (images). To make the best of our limited number of bacteria images, we used data augmentation and transfer learning. CNN and ResNet have feature learning capabilities such as curves, lines, or more complex topological features. Many of the features are common regardless of the objects in the images that these models were trained on. So, even if ImageNet contains no cell images, the features that the models learned are to a varying degree useful. In brief, in this study, we use residual networks pre-trained on the ImageNet database (Deng et al., 2009) to classify longitudinal division bacteria.

## 2. Methods

**Figure 3:**
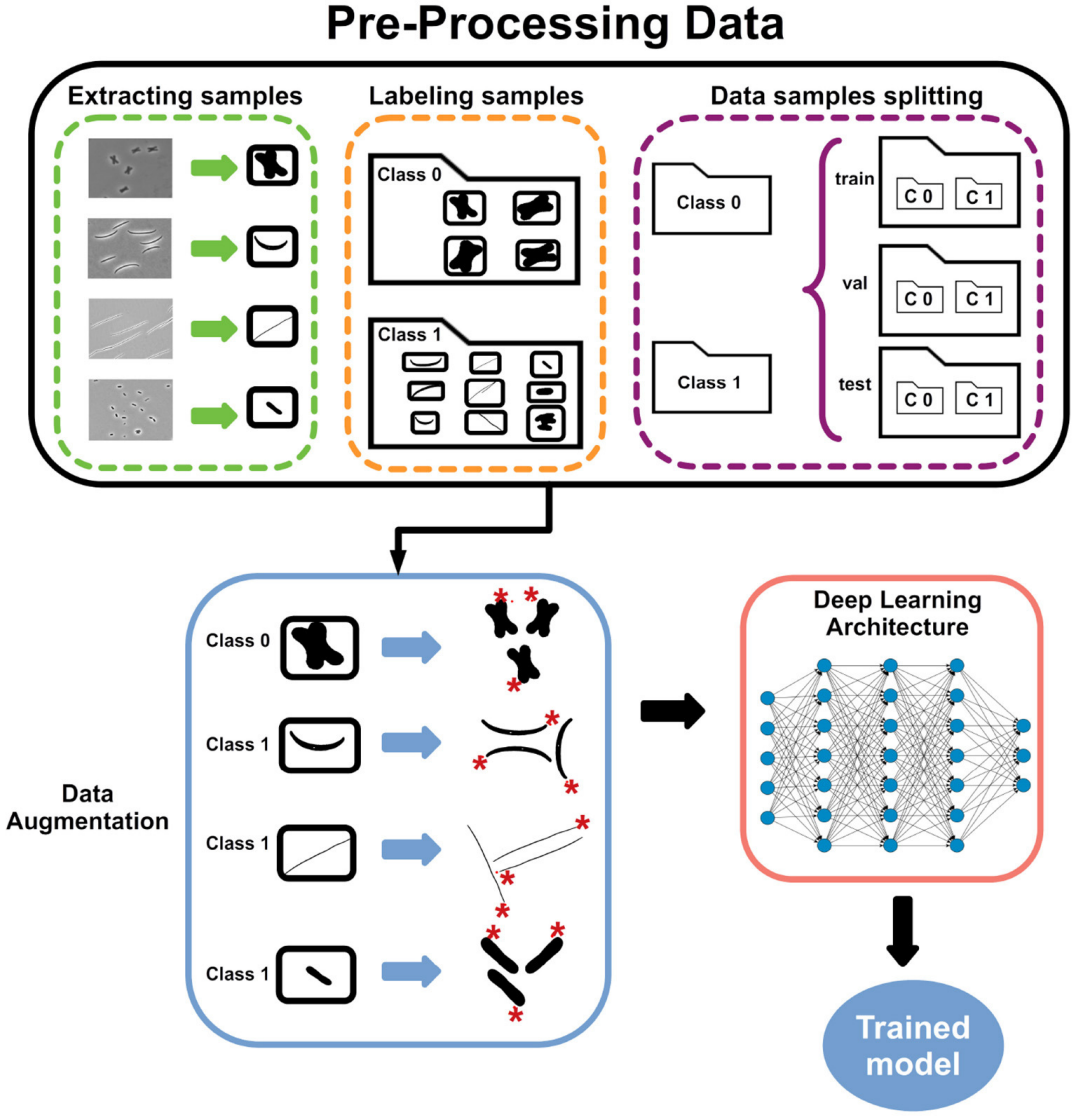
Proposed pipeline. The first part prepares the dataset by pre-processing the images for both classes. The pre-processing step extract into single image files each microscopic image, thus, creating a new images dataset. Then, the images dataset is divided into three sub-datasets: training, validation, and testing. Internally PyTorch will label the content of the folders train and validation into Class 0 (longitudinal_division) and Class 1 (other_division). Next, data augmentation is applied by rotating, scaling, and normalizing each image. Finally, the deep learning model is trained using the transfer learning technique.

In this section, we introduce the training pipeline for the classification of longitudinal division bacteria based on microscopic images. In our study, we performed a binary classification of bacteria division images. Hence, the image dataset contains two types of classes: “longitudinal division” and “other division.” In order to obtain the training samples, we extract the sub-images from 730 microscope images, 468 for the first class and 262 for the second class. However, to avoid manually extracting the samples from microscope images, we use an in-house software (Schreiber et al., 2021). Finally, with the complete dataset, we used a pre-trained deep learning CNN to estimate the best model to classify longitudinal division. Figure 3 shows the proposed pipeline. All the steps of the process are detailed below in the rest of the section.

### 2.1. Dataset

#### 2.1.1. Pre-processing: Extraction and Selection of Samples

The dataset is a collection of phase contrast microscopic images showing bacteria with a variety of shapes that were manually labeled. The microscopic images are from the Thiosymbion species extracted from shallow-water sediments in Belize. More details about the images can be found in Pende et al. (2014), Leisch et al. (2016), and Weber et al. (2019). Each microscopic image contains only a single bacteria cell shape. Each image was later split into individual bacteria images.

**Figure 4:**
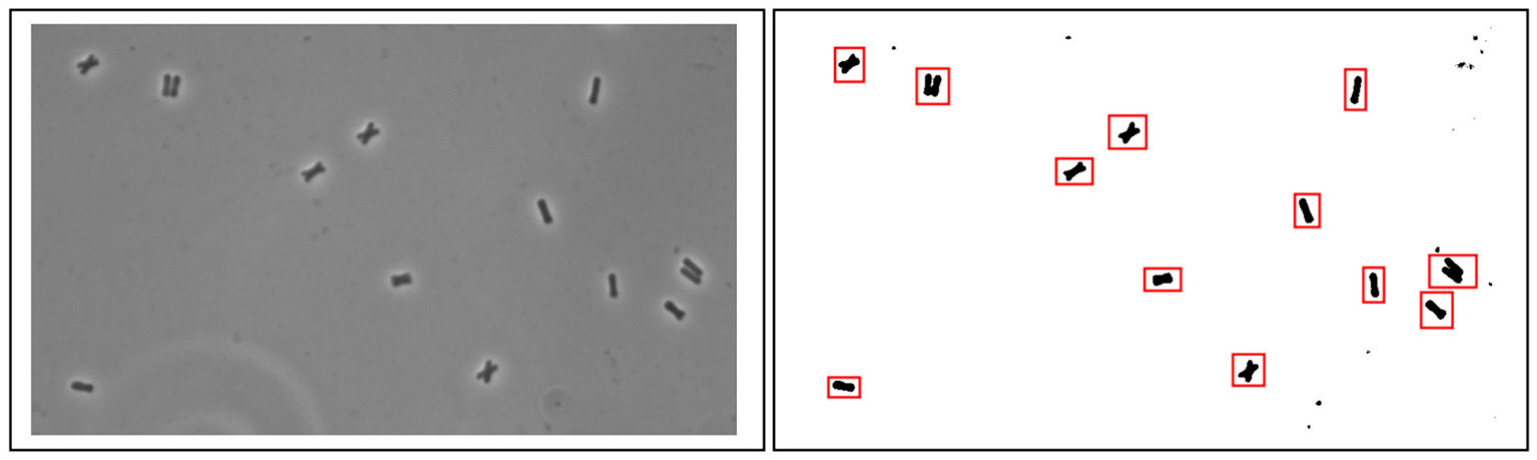
As an alternative to manual extraction of samples from the microscopic images, the pre-processing was done with our in-house software to create the datasets for training, validation, and testing. The tool transforms the image into black and white to be able to extract images of bacteria. We have added the red boxes to the reader to see the areas that our tool marks as potential bacteria. After running our tool, we had to visually curate the image results.

The steps are as follows: we start with microscopic images of the “longitudinal division” class and then with the “other division” class. All microscope images from one class were put in the same folder. Next, we use an in-house software (Schreiber et al., 2021) to perform the following tasks: (1) transform each image into a black and white image, (2) label contiguous areas of black pixels as a group (representing one bacteria), (3) then each group is bounding-boxed. In other words, the tool saved the pixels' coordinates to extract the image of the bacteria. This process can be performed manually or with other software. Figure 4 shows the representation of the bounding box. The procedure ignores small areas according to a threshold selected a priori. Finally, each group is exported to a PNG file image format. We run the in-house tool separately for each class.

#### 2.1.2. Data Partitioning

The dataset contains a total of 15,090 bacteria images, where 2,244 and 12,846 belong to the class “longitudinal division” and “other division,” respectively. We randomly divided the dataset into three subsets: train, validation, and testing with Scikit-learn (Pedregosa et al., 2011) library. First, 33% of the dataset was kept for the testing set. The remaining 66% was further split into 80% for training and 20% for validation.

The training data are used to compute the updates of the model weights. The purpose of validation is to reduce the error rate during the training by predicting the accuracy of the model. This is useful for the user to tune the training hyperparameters. Test sets are not used in the training and are only used to compute the performance measures after the training. It is possible to have a second code to run a test. For both classes, the subsets had the following number of samples shown in Table 1.

**Table 1:** Number of images in the split dataset.

	**Training**	**Validation**	**Testing**	**Total**
Longitudinal division	1,202	301	741	2,244
Other division	6,884	1,722	4,240	12,846

### 2.2. Training

PyTorch (Paszke et al., 2019) is a recent deep learning framework that provides implementations to build CNNs and is highly demanded in computer vision tasks. We use PyTorch version 1.5 for CUDA version 10.2 to run on a graphic processing unit (GPU). The complete list of python libraries can be found in the link to the repository mentioned in the Supplementary Data section.

We run the code in a cloud service and in our local cluster. We have used the services provided in DEEP Hybrid DataCloud (López García et al., 2020) for the cloud, namely DEEP-as-a-Service API (DEEPaaS) and Dashboard (a web interface to a cloud of hardwares) to deploy our application as a Docker container. DEEPaaS furnishes the graphical user interface on a browser from which to trigger the training. We also had access to console-like interface via Jupyterlab to debug directly inside the remotely deployed container. The node to which we deployed our training had NVIDIA V100 GPU with 32 GB RAM. The running time for 25 epochs was approximately 20 min including container deployment and data transfer to a cloud hardware. For our local cluster, we had Intel Xeon Platinum 8280L CPU 2.70 GHz, System RAM 1.5 TB, GPU Nvidia V100-SXM3-32 gb and GPU RAM: 32 GB, and CentOS Operating System.

In this study, we use a pre-trained CNN ResNet-18 from PyTorch to classify bacteria division types by means of transfer learning. As described previously, we pre-processed the images applying data augmentation for the training and testing dataset. Due to the different sizes of the images, width between 35 and 4,295 pixels and height between 35 and 6,185 pixels, all images were resized to 128 × 128 pixels then randomly rotated, followed by random horizontal and vertical flips following the PyTorch recommendation. We train the model with the optimizer, Stochastic Gradient Descent (SGD) (Bottou, 2010). A short description of the algorithm can be found in the next section. A training setup must consider different hyperparameters. A typical set of hyperparameters are as follows: number of iterations over the entire training samples (called epochs), a batch size which is the number of samples used in one update of weights, and learning rate that controls the convergence of optimization. For the model trained in the current study, the training hyperparameters were set to: 25 epochs, batch of 16 images, 0.001 learning rate, 0.9 momentum factor (equation 13), 7 epoch-period of learning rate decay, and 0.1 multiplicative factor of learning rate decay. Our Python script made use of Numpy (Harris et al., 2020) and Scikit-Learn (Pedregosa et al., 2011).

### 2.3. Optimization Algorithm

A machine learning method employs an optimization algorithm to minimize its prediction error. In neural networks, it is easy to obtain gradient information. Furthermore, the search space can be very high dimensional for DNNs, hence the popularity of gradient-based optimization methods in this field. The efficiency of stochastic gradient descent in large-scale machine learning is documented in Bottou (2010). Here, we briefly describe the method to aid in the understanding of the definition of the hyperparameters mentioned in the previous section. Let us denote the objective (loss) function as *J*, which depends on the data *X* with a large number of input–output pairs (say *N* pairs in total) and parameters θ that we can control to minimize the loss *J*. Equation 10 describes the recursive formula with which gradient descent algorithm approaches θ that minimizes the loss *J*.

(10) $$\begin{array}{r} \theta_{t+1}=\theta_{t}-\alpha \cdot \nabla_{\theta }J\left(X,\theta_{t}\right), \end{array}$$

where *t* is the iteration counter, α is an adequately chosen learning rate, and ∇_θ_ denotes the gradient with respect to θ.

In stochastic gradient descent, the parameters θ are updated with every input–output pair in *X*. Thus, we have at iteration *t*,

(11) $$\begin{array}{r} \theta_{t+1}=\theta_{t}-\alpha \cdot \nabla_{\theta }J\left(X^{\left(i\right)},\theta_{t}\right), \end{array}$$

where *X*^(*i*)^ denotes an instance of input—output pair in the data *X*, and *i* ∈ {1, 2, …, *N*} is the index for the input—output pair of *X* whose total number of pairs counted as *N*.

A mini-batch approach can typically be taken in which the gradient is calculated as an average of small subset of training data,

(12) $$\begin{array}{r} \theta_{t+1}=\theta_{t}-\alpha \cdot \frac{1}{\left|B\right|}\underset{i\in B}{\sum }\nabla_{\theta }J\left(X^{\left(i\right)},\theta_{t}\right), \end{array}$$

where *B* denotes a random subset of {1, 2, …, *N*} of the training data, and |*B*| denotes the cardinality (number of elements) of the set. An epoch completes having chosen all *N* pictures by removing |*B*| images after each iteration. In PyTorch, momentum factor is introduced to modify the above into two-step computation

(13) $$\begin{array}{r} v_{t+1}=\mu \cdot v_{t}+\alpha \cdot \frac{1}{\left|B\right|}\underset{i\in B}{\sum }\nabla_{\theta }J\left(X^{\left(i\right)},\theta_{t}\right), \end{array}$$

(14) $$\begin{array}{r} \theta_{t+1}=\theta_{t}-v_{t+1}. \end{array}$$

where μ is the momentum factor and *v* is a velocity vector influenced by gradients of previous steps. In PyTorch you also find a period of learning decay and a factor of learning decay. That is, a factor is multiplied to α every number of epochs in the training cycle. After every fixed number of epochs, the algorithm performs a substitution α = γα, where γ is the factor of learning decay. The period and the factor of learning decay are set by the user.

## 3. Results

In this section, we describe the training results and the evaluation of the model for the effectiveness of the proposed method. Moreover, we train another model without the pre-trained network to compare the performances using transfer learning. The results are shown in the following.

Table 2 shows the mean and standard deviation of training time in our local GPU cluster. Five training runs were performed. Supplementary Material describes the statistics of the trained models' performances. The code can train without a GPU but with a longer time of running.

**Table 2:** Training times in seconds for pre-trained and non pre-trained ResNet-18.

	**25 epochs**	**35 epochs**	**100 epochs**
pre-trained network	397.6 ± 1*s*	557.8 ± 3*s*	1596.6 ± 10*s*
non pre-trained	393.4 ± 3*s*	554 ± 4*s*	1587.2 ± 12*s*

*The mean and standard deviation are calculated from five runs*.

**Figure 5:**
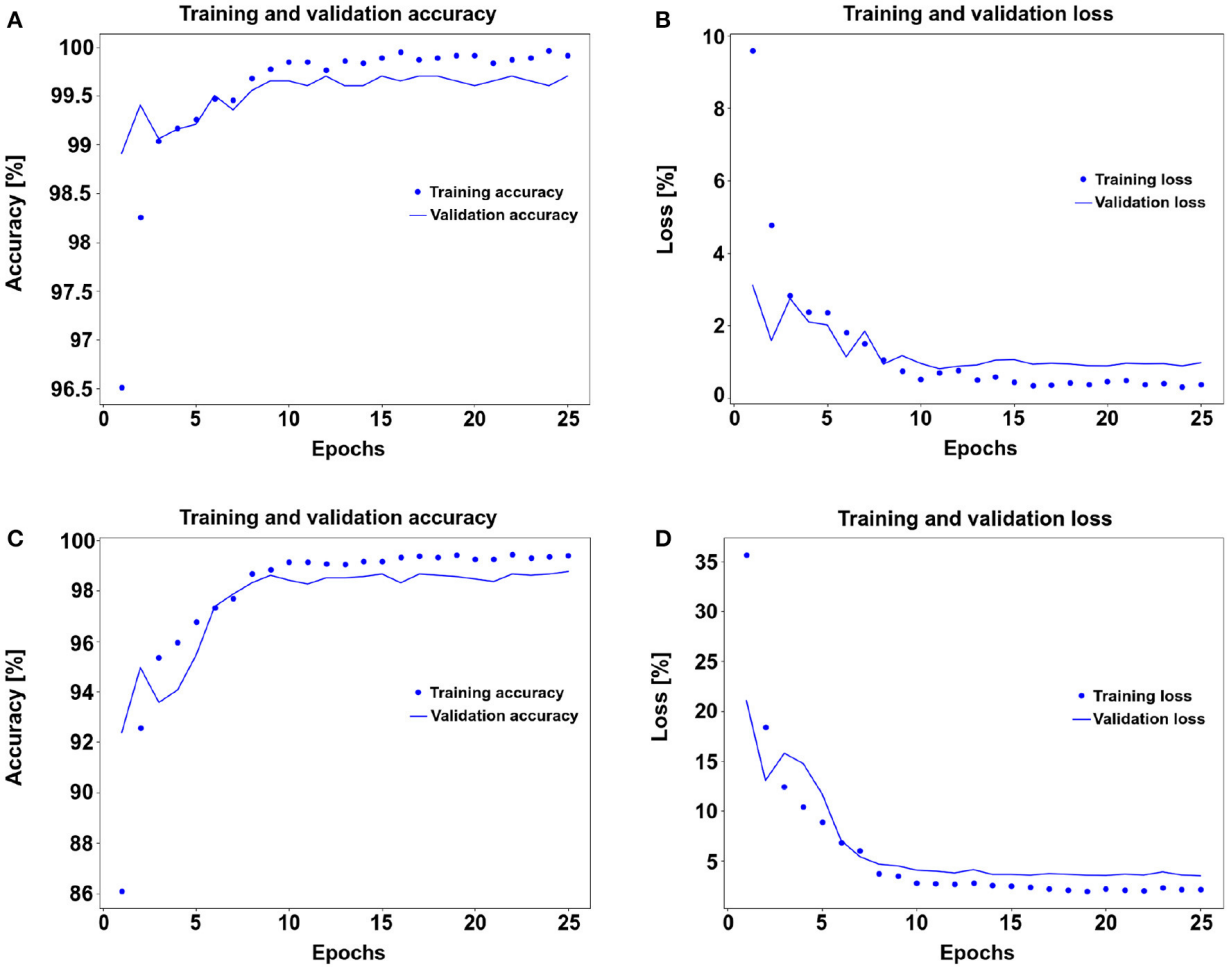
Curve plots showing the training and validation for accuracy and loss from the pre-trained convolutional neural network (CNN) ResNet-18 **(A,B)** and without a pre-trained network **(C,D)** for 25 epochs each. **(A)** The training starting at 96.5% and stabilize around the 11 epoch, while the validation starts at 99% and stabilize around epoch 10. **(B)** The training loss starts at 10.0% decreasing gradually until stabilize at epoch 10, the validation starts at 3% and stabilizes at epoch 11. **(C)** The training starts at 86% and stabilizes at epoch 10 with 99% score and the validation around 92% and stabilizes at 98% in epoch 10. **(D)** The loss for the training starts at 35% and the validation at 20% both stabilize at epoch 10 with scores around 5% for validation and training around 4%.

**Figure 6:**
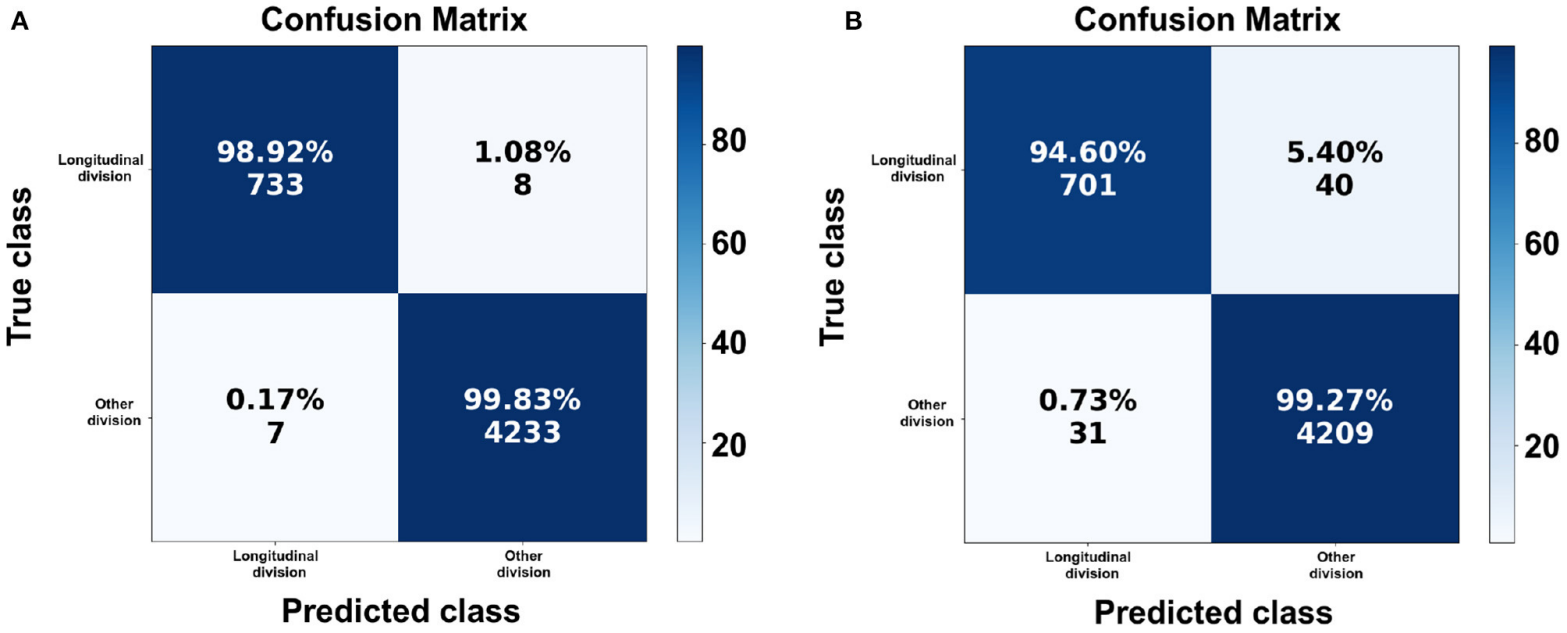
The confusion matrices for the classification of longitudinal division bacteria. The confusion matrix for the pre-trained convolutional neural network (CNN) ResNet-18 **(A)** and without a pre-trained network **(B)**. **(A)** Note that 733 of 741 images are correctly classified and **(B)** 701 of 741 images are correctly classified.

For the first model with a pre-trained network, the accuracy score for the test set was 99.6988% while the best validation accuracy was 99.7528%. Figures 5A,B show the accuracy and error histories of the training set and validation set with respect to the number of epochs. The testing loss was 0.6639%. The confusion matrix in Figure 6A shows that 98.9203% of the longitudinal division bacteria were classified correctly by the pre-trained ResNet-18.

In the second model, without the pre-training, the accuracy score of the test set was 98.5745%, the best validation accuracy was 98.7642%. The testing loss accuracy was 3.9564%. The learning and error curves are shown in Figures 5C,D, respectively. Finally, the confusion matrix shown in Figure 6B shows that 94.6018% of the longitudinal division bacteria were classified correctly.

**Figure 7:**
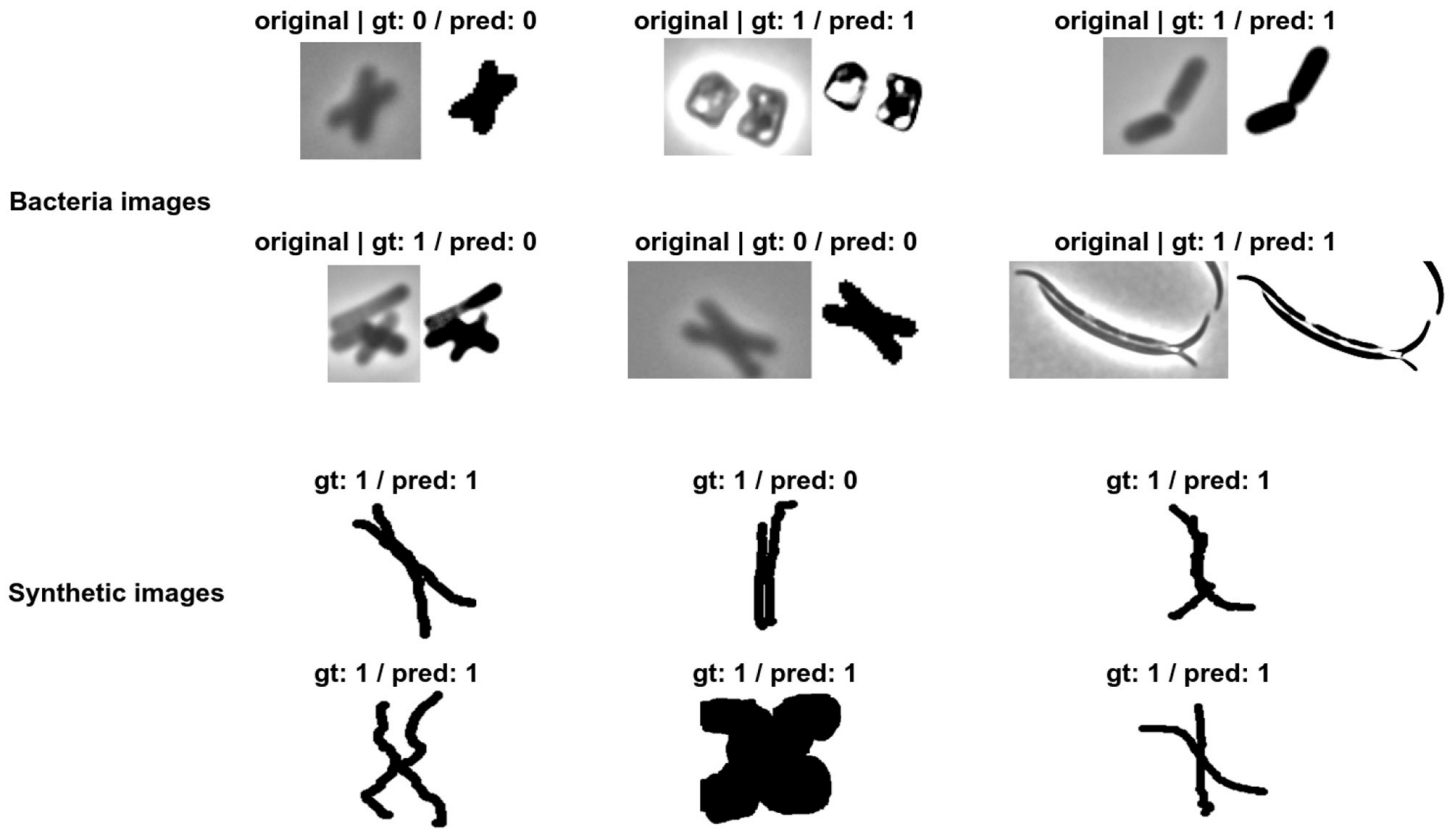
Prediction of class on unseen images. We test the accuracy of the trained model with two types of samples, real images from the test folder and synthetic images draw by hand. On the left side of each prediction, we show the original image extracted from the microscopic image, followed by the ground truth (gt) and the prediction class (pred). Class 0 is longitudinal division and class 1 is other divisions.

Figure 7 shows prediction examples on unseen cell images. It shows different morphologies and corresponding predictions by the trained model for both real and synthetic cell images. The synthetic images are adversarial in the sense that they try to fool the model to make false positives. The synthetic images were created by hand, and the purpose was to understand what would cause the model to miss classify. It may merit a further investigation on optimally performing the generation of adversarial images. However, this would require another research on its own and this is out of the scope of the current paper. The middle image in the third row is an example of a false positive, suggesting that a very narrow crease may be interpreted as a longitudinal split.

### 3.1. Performance Metrics

Besides the accuracy and the loss, we also look into recall, precision, and F1 to measure the performance of the trained models. Let us denote the two classes as positive and negative in a binary classification problem. Precision measures the ratio of how many of the predicted positives (true positives and false positives) were actually positive (true positives). That is,

$$P=\frac{\text{TP}}{\text{TP}+\text{FP}},$$

where TP denotes the number of true positives, FP denotes the number of false positives, and P denotes the precision. Recall measures the ratio of how many of the actual positives (true positives and false negatives) were predicted correctly as positives (true positives),

$$R=\frac{\text{TP}}{\text{TP}+\text{FN}},$$

where FN denotes the number of false negatives and R denotes recall. F1 (Dice, 1945) is computed using precision (P) and recall (R) as

$$\text{F1}=2\frac{P\cdot R}{P+R}$$

F1 is 1 when there is neither FP nor FN, and 0 when there is no TP. F1 is particularly useful when the number of positive and negative classes are substantially different or imbalanced in the data.

Table 3 shows the performance of the proposed method for longitudinal division classification for the pre-trained CNN ResNet-18. We can see that the precision was 99.0541%, the recall was 98.9204%, and the F1 score was 98.9872%. The class “Other division” performed as follows: the precision was 99.8114%, the recall was 99.8349%, and the F1 score was 99.8231%. We can corroborate these scores with Figure 6A that for class “longitudinal division” 733 of 741 were predicted correctly and for the class “other division” 4,233 of 4,240 were predicted correctly. Table 4 shows the performance for the model without a pre-trained network.

**Table 3:** Metrics for the pre-trained convolutional neural network (CNN) ResNet-18.

	**Precision**	**Recall**	**F1-score**
Longitudinal division	99.0541%	98.9204%	98.9872%
Other division	99.8114%	99.8349%	99.8231%

**Table 4:** Metrics for the model without a pre-trained network.

	**Precision**	**Recall**	**F1-score**
Longitudinal division	95.7650%	94.6019%	95.1799%
Other division	99.0586%	99.2689%	99.1636%

## 4. Discussion

The results show that automatic identification of longitudinal division is possible using ResNet. The use of transfer learning has been successfully applied in the past to detect, count, and classify cells. For instance, U-Net (Falk et al., 2019) is a tool based on deep learning that segments biomedical images. This tool employs models previously trained with U-Net to segment unseen images. In our case, we do not have models previously trained in our classification problem; however, we were able to exploit the advantages of a general model like ResNet to successfully classify the longitudinal division of bacteria. We must highlight that this is the first time that the DL was applied to this classification problem.

The pre-trained model shows high test accuracy from the beginning and by epoch 5 (79.86±0.573 s) the accuracy is more or less stabilized at 99%. On the other hand, the non-pre-trained model stabilizes at around epoch 12 (190.46±1.549 s). In the case of the pre-trained model, we could have run for fewer epochs, say 12, and still get essentially the same predictive performances. The losses confirm the same tendency: the pre-trained model stabilizes at epoch 5 and the non-pre-trained model stabilizes at epoch 12.

Some non-transfer-learning-based methods for classifying bacteria focus on different types of features that go beyond the geometrical shape, taking into account other features such as brightness and contrast, color, or the way bacteria arrange (Zieliński et al., 2017; Mohamed and Afify, 2018). However, for these methods, it is required to extract the features prior to classification with support vector machine (SVM). This is because they do not have enough microscopic images for training the DNNs. Therefore, it was necessary to first identify few features that are effective in classifying the classes of bacteria and use classical ML.

In our proposed approach, we split the microscopic image into single bacteria images and then apply data augmentation. This way we can perform the classification without worrying about feature extraction. Moreover, we can benefit from the pre-trained network (transfer learning), which minimized the computational cost of learning the classification task.

We have had only one set of training, validation, and test data thus far. However, we run five times the training with different sets of epochs, the results are shown in the Supplementary Material. Nevertheless, it would be desirable to further investigate the predictive performance by training on different data as well as investigating the predictive performances using different initialization of weights and model architecture.

## 5. Conclusion

Although manual labeling of the bacteria division type is time consuming, our study shows that automation of classification of bacteria division type can be very accurately predicted with available data. As future work, we plan to add into the pipeline the image segmentation from our in-house tool to label the bacteria over the microscopic image.

Our development enables fast and automatic identification of bacteria images and discriminating longitudinal bacteria fission. The pre-trained model can expedite the training requiring fewer epochs due to already learned image features. This deep-neural-network-based approach has the potential to be applied to other exotic morphological features and can be useful when we have a large set of images for cell counting or determining division rate.

## Data Availability Statement

The code and the datasets presented in this study can be found in the online GitHub repository: https://github.com/charlos1204/longitudinal_division_classification.git. Further inquiries can be directed to the corresponding authors.

## Author Contributions

CG-P, KI, and RF wrote the deep learning code. CG-P and KI constructed models. NS wrote the code for extracting the samples from the microscopic images. JG, CG-P, and KI analyzed the data and made the figures. All authors wrote and revised the manuscript.

## Conflict of Interest

The authors declare that the research was conducted in the absence of any commercial or financial relationships that could be construed as a potential conflict of interest.
