# Supplementary material for: Efficient Detection of Longitudinal Bacteria Fission Using Transfer Learning in Deep Neural Networks
Source: Front Microbiol. 2021 Jun 8;12:645972. doi: 10.3389/fmicb.2021.645972 (PMC8217615; doi:10.3389/fmicb.2021.645972)
Supplement: Supplementary file 1 [file Data_Sheet_1.PDF]

# Supplementary Material

## 1 SUPPLEMENTARY DATA

Below we provide results of the five training runs that were performed on our local cluster with GPU Nvidia V100-SXM3-32gb and GPU RAM: 32 GB. All five runs used the same training, validation and test data. The hyperparameter settings were also the same for all the runs. Thus, the difference between the runs is due to the stochastic nature of the training algorithm. The five training runs were performed for different numbers of termination epochs, namely, 25, 35, and 100.

Table S1 shows the accuracy reached by the pre-trained and non pre-trained model at fixed numbers of epochs.

| | **25 epochs** | **35 epochs** | **100 epochs** |
|---|---|---|---|
| pre-trained | $99.6065\% \pm 0.071830\%$ | $99.6145\% \pm 0.076951\%$ | $99.6466\% \pm 0.111428\%$ |
| non pre-trained | $98.5304\% \pm 0.124893\%$ | $98.4340\% \pm 0.173272\%$ | $98.4621\% \pm 0.089358\%$ |

**Table S1.** Accuracy score of the pre-trained and non pre-trained ResNet-18 for 25, 35 and 100 epochs. The mean and standard deviation are calculated from five runs.

Table S2 shows precision, recall, and F1 scores for the pre-trained network. Similarly, Table S3 shows the same performance metrics for the non pre-trained model.

| | **Precision** | **Recall** | **F1-score** |
|---|---|---|---|
| 25 epochs | $98.3660\% \pm 0.347111\%$ | $99.0013\% \pm 0.585129\%$ | $98.6812\% \pm 0.243472\%$ |
| 35 epochs | $98.7068\% \pm 0.480478\%$ | $98.7044\% \pm 0.411545\%$ | $98.7045\% \pm 0.257231\%$ |
| 100 epochs | $98.8131\% \pm 0.408760\%$ | $98.8124\% \pm 0.490307\%$ | $98.8122\% \pm 0.375777\%$ |

**Table S2.** Metrics for Longitudinal division class (Class 0) with the pre-trained ResNet-18 for 25, 35 and 100 epochs. The mean and standard deviation are calculated from the five runs.

| | **Precision** | **Recall** | **F1-score** |
|---|---|---|---|
| 25 epochs | $95.9393\% \pm 0.772058\%$ | $94.1160\% \pm 1.25005\%$ | $95.0114\% \pm 0.450709\%$ |
| 35 epochs | $95.8173\% \pm 1.06794\%$ | $93.5762\% \pm 1.47093\%$ | $94.6725\% \pm 0.609215\%$ |
| 100 epochs | $95.2181\% \pm 0.701724\%$ | $94.4129\% \pm 0.985230\%$ | $94.8085\% \pm 0.320339\%$ |

**Table S3.** Metrics for Longitudinal division class (Class 0) with the non pre-trained ResNet-18 for 25, 35 and 100 epochs. The mean and standard deviation are calculated from the five runs.

In all the performance measures ( accuracy, precision, recall, and F1-score), we see that the pre-trained models perform consistently better than non pre-trained models. However, both pre-trained and non

pre-trained models showed very good performances. The difference between the runs was small, mostly manifesting only from the third significant digits and on.

Figure S1 and S2 show the history plots of the accuracy and the loss of five training runs for pre-trained (left column) non pre-trained models (right column). The dotted lines indicate accuracy for the training data and the solid ones indicate the accuracy for the validation data. Up to 100 epochs, we did not observe obvious degradation in validation performances. The zig-zag nature of the solid lines comes from the fluctuations in the third significant digits. The performance of pre-trained models is very high from the start compared to the non pre-trained models. Likewise, the loss for the pre-trained model starts very low at around 10% and for the non pre-trained model, starts higher at around 35%.

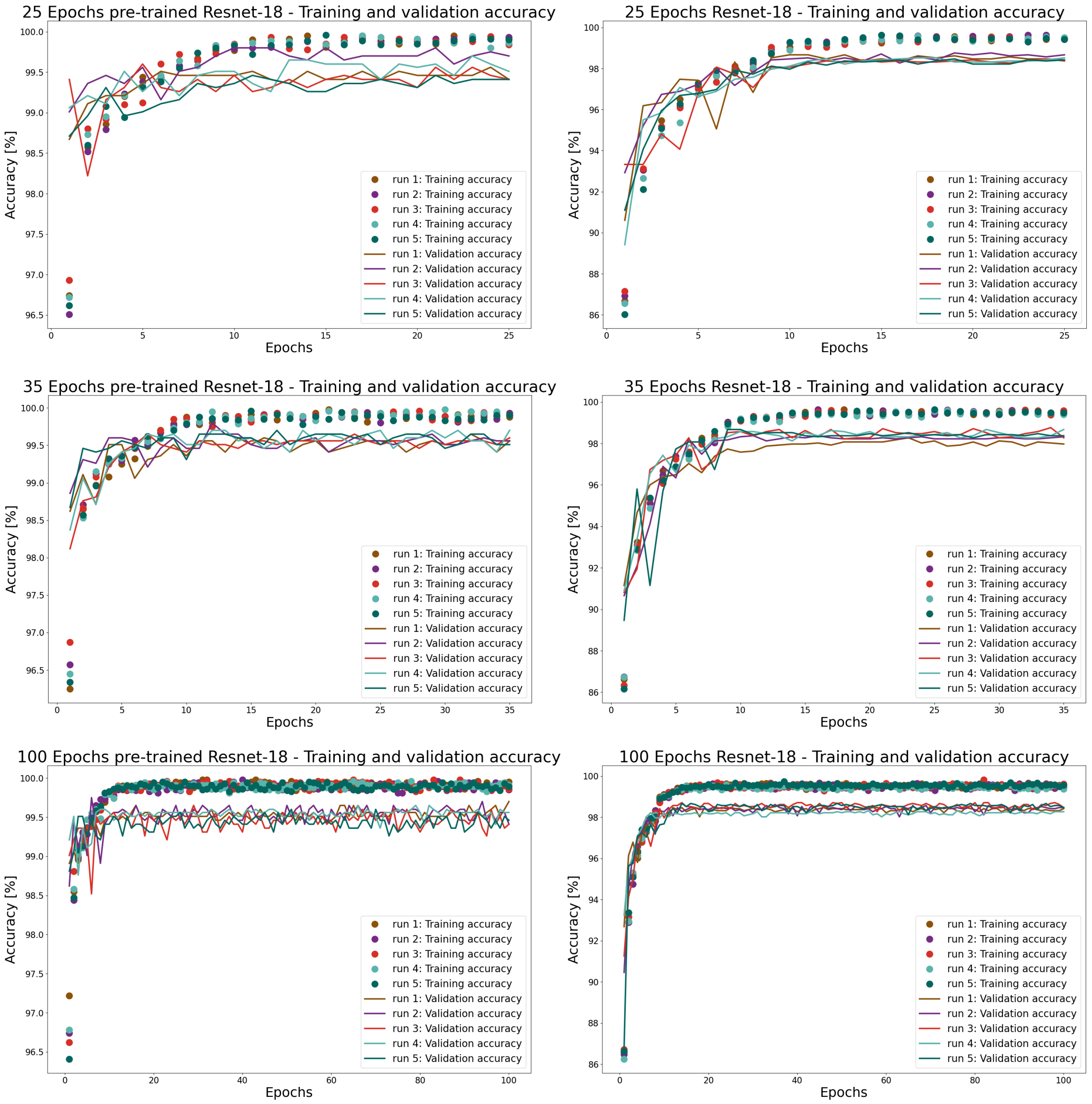


**Figure S1.** Training and validation accuracy curves of the models with pre-trained ResNet18 vs non pre-trained ResNet18. The training run for 25, 35 and 100 epochs.

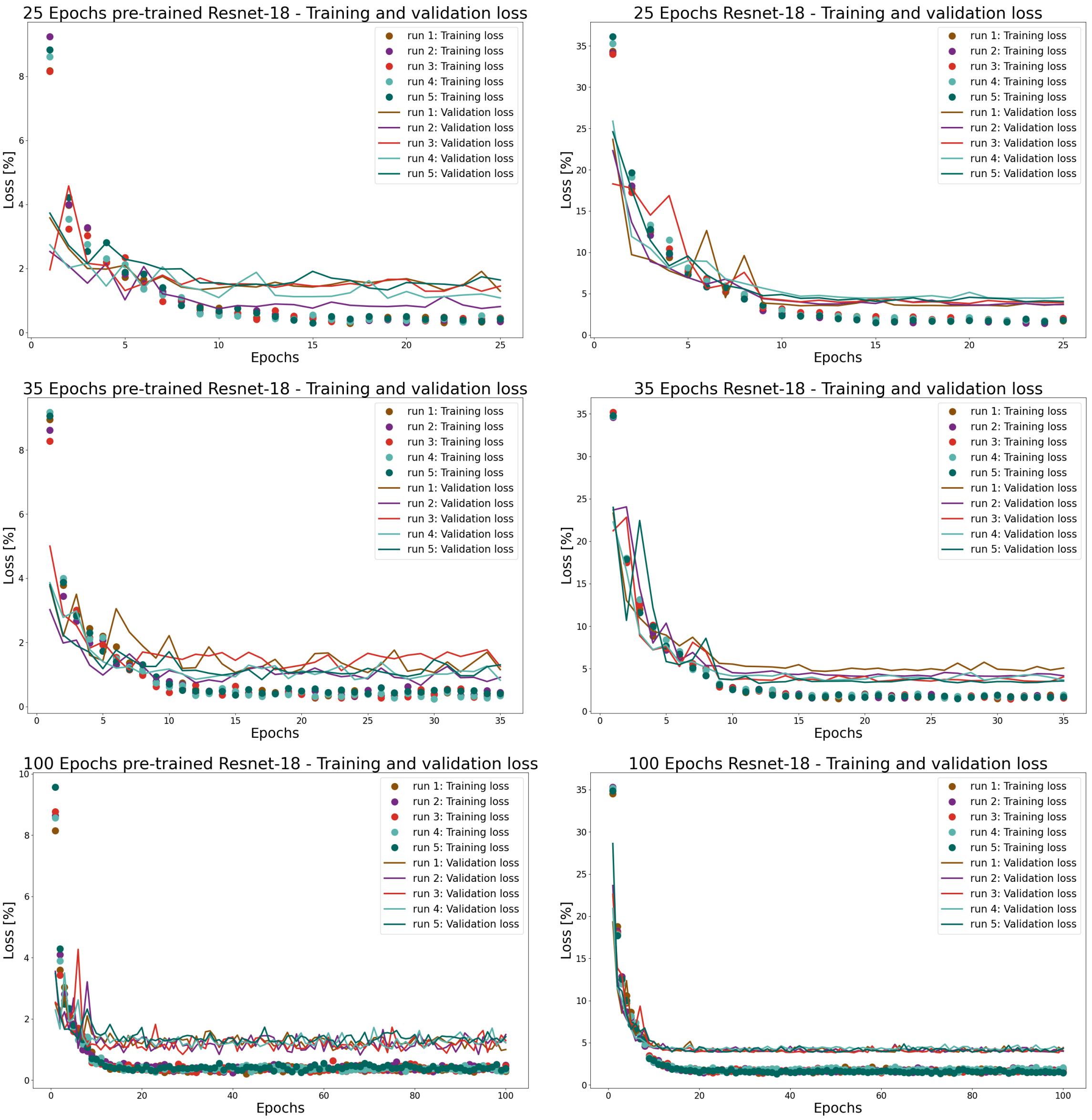


**Figure S2.** Training and validation loss curves of the models with pre-trained ResNet18 vs non pre-trained ResNet18. The training run for 25, 35 and 100 epochs.
